# Photocatalytic Aerobic Oxidation of Biomass-Derived 5-HMF to DFF over MIL-53(Fe)/g-C_3_N_4_ Composite

**DOI:** 10.3390/molecules27238537

**Published:** 2022-12-04

**Authors:** Danyao Huang, Hao Wang, Ying Wu

**Affiliations:** Key Laboratory of the Ministry of Education for Advanced Catalysis Materials, Institute of Physical Chemistry, College of Chemistry and Life Science, Zhejiang Normal University, Jinhua 321004, China

**Keywords:** photocatalysis, aerobic oxidation, MIL-53(Fe)/g-C_3_N_4_, heterojunction composite

## Abstract

A MIL-53(Fe)/g-C_3_N_4_ heterogeneous composite was synthesized and applied in photocatalytic oxidation of 5-hydroxymethylfurfural (5-HMF) to 2,5-diformylfuran (DFF). The systematic investigation indicated that the introduction of MIL-53(Fe) into g-C_3_N_4_ increased the specific surface area, broadened the visible-light response region, and promoted the separation efficiency of the photo-generated electron-hole pairs. The 10% MIL-53(Fe)/g-C_3_N_4_ heterogeneous composite achieved the best photocatalytic oxidation activity with 74.5% of 5-HMF conversion under simulated sunlight, which was much higher than that of pristine g-C_3_N_4_ and MIL-53(Fe). The MIL-53(Fe)/g-C_3_N_4_ composite displayed good photocatalytic reusability and stability. Based on the characterization results and photocatalytic performance, a Z-scheme photocatalytic mechanism of the MIL-53(Fe)/g-C_3_N_4_ composite was suggested, and a possible reaction route was deduced.

## 1. Introduction

The development and utilization of abundant renewable biomass resources to synthesize high-value-added chemicals is one of the effective ways to solve the energy problem. 5-Hydroxymethylfurfural (5-HMF), as an important biomass platform compound, can be converted into a variety of oxidation products, such as the high-value-added product 2,5-diformylfuran (DFF) [1]. DFF has wide applications not only as a monomer for polymerized materials, but also as an important chemical intermediate for the synthesis of fungicides, drugs, heterocyclic compounds, and functional polymers [2,3]. At present, DFF is mainly synthesized via the chemical selective oxidation of 5-HMF. However, the request of high temperature, high oxygen pressure, and expensive noble metal makes this reaction high energy consuming and environmentally unfriendly [4,5,6,7,8].

In comparison, the photocatalytic technique has been widely used with various semiconductor-based photocatalysts due to the mild reaction using solar light at room temperature [9,10]. In recent years, the photocatalytic oxidation of alcohols to the corresponding aldehyde has received more and more attention. However, DFF is not easy to obtain with high selectivity and yield because 5-HMF has multiple active functional groups. The most used photocatalysts for 5-HMF oxidation include metal oxides and metal-free g-C_3_N_4_-based composites. In 2013, Yurdakal et al. [11] first used self-made TiO_2_ to catalyze 5-HMF into DFF under ultraviolet light. However, the formed ·OH species had a strong oxidizing property, leading to low DFF selectivity due to the deep oxidation of 5-HMF. Exploring a visible-light responsive catalyst for the efficient selective conversion of 5-HMF to DFF is challenging and desirable. Although thereafter the modified TiO_2_ [12,13,14,15], Nb_2_O_5_ [16], MnO_2_ [17], and ZnO/PPy [18] photocatalysts were successively used for this reaction, metal-free g-C_3_N_4_ is more attractive because it is low cost, easy to obtain, and exhibits promising photocatalytic properties due to its heptazine ring structure and delocalized conjugated π structures [19,20]. In addition, g-C_3_N_4_ is attractive because its visible-light responsiveness and its narrow band gap energy (~2.7 eV) makes a semiconductor. Furthermore, the relatively negative conduction band (CB) position (−1.19 eV) leads to its high reduction ability of photo-generated electrons. At the same time, the valence band (VB) position at 1.59 eV makes it unable to generate non-selective hydroxyl radical, which is beneficial to inhibiting the deep-oxidation of alcohols and improving the selectivity of aldehyde. These characteristics make g-C_3_N_4_ a promising photocatalyst for the selective oxidation of 5-HMF. Krivtsov et al. [21] used the high-temperature thermos-exfoliated method to prepare g-C_3_N_4_ for the oxidation of 5-HMF. The DFF selectivity of 30–50% can only be obtained under ultraviolet light. By contrast, the results reported by Wu et al. [22] showed that both ultraviolet light and visible light can induce self-made g-C_3_N_4_ to catalyze the oxidation reaction. Nevertheless, the photocatalytic efficiency of g-C_3_N_4_ was lower than anticipated due to a high carrier recombination rate. The loading of NaNbO_3_ [23] and cobalt thioporphyrazine (CoPz) [24] can enhance the reaction activity of g-C_3_N_4_ for the oxidation of 5-HMF. Nevertheless, the obtained product was not DFF, but 2-formyl-5-furancarboxylic acid (FFCA) and 2,5-furandicarboxylic acid (FDCA), respectively. By compounding WO_3_ [25] or BiWO_3_ [26] with g-C_3_N_4_ to construct heterojunction, the photocatalytic performance can be remarkably improved. However, the pristine WO_3_ and BiWO_3_ semiconductors showed negligible reactive activity and only played the role of promoting the separation of photo-generated charges. Therefore, it is desired to explore a reactive semiconductor to construct a heterojunction composite with g-C_3_N_4_, which is more conductive to improving the reaction performance for the oxidation of 5-HMF to DFF.

MIL series metal-organic frameworks (MOFs) are competent as a type of efficient photocatalysts because of nontoxicity, low cost, and high chemical stability [27,28,29,30,31,32]. MIL-53(Fe) is an Fe-containing MOF with a narrow band gap of ~2.5 eV and is considered a promising visible-light-responsive semiconductor photocatalyst because the existence of extensive oxo-iron (Fe-O) clusters makes it have prominent optical properties. In addition, it has high hydrothermal stability and strong acid resistance. Despite these advantages, the photocatalytic performance of MIL-53(Fe) is restricted by the fast recombination of photo-generated electrons and holes, resulting in a low quantum conversion efficiency [33,34]. The reaction activity of MIL-53(Fe) can be improved via the combination with g-C_3_N_4_ by using the interface effect. The formed MIL-53(Fe)/g-C_3_N_4_ composite has been used for photocatalytic hydrogen production and Cr(IV) reduction [35,36]. To the best of our knowledge, however, the composite has not yet been applied in the selective oxidation of 5-HMF to DFF.

In this paper, the Z-scheme MIL-53(Fe)/g-C_3_N_4_ heterojunction composites were fabricated using a hydrothermal method. In the Z-scheme photocatalytic system, the separation efficiency of photogenerated carriers were improved via the recombination of the low reactive charge carriers at the interface while retaining strong redox species. The photoinduced e^−^ in CB of g-C_3_N_4_ presents strong reducibility and h^+^ in VB of MIL-53(Fe) exhibits good oxidation capacity. Then, the composites were applied to photocatalytic oxidation of 5-HMF to DFF. The effect of different ratios of MIL-53(Fe) to g-C_3_N_4_ on the photocatalytic performance was investigated. The physicochemical properties were thoroughly characterized. The charge transfer mechanism of the photocatalyst and the reaction route were deeply discussed. This study affords an effective strategy for selective oxidation of biomass-derived compound based on high performance Z-scheme photocatalysts with solar light response.

## 2. Results and Discussions

### 2.1. Catalyst Characterizations

#### 2.1.1. Structure Characterization

**Figure 1:**
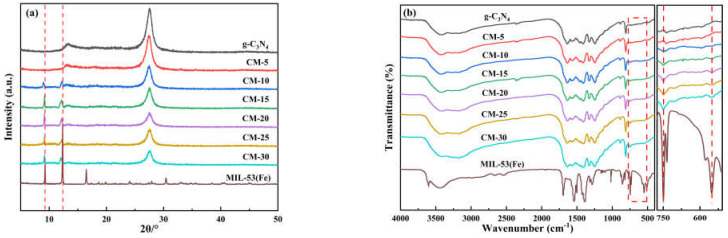
(**a**) XRD patterns and (**b**) FT-IR spectra of MIL-53(Fe)/g-C_3_N_4_ samples.

The structure of the as-prepared MIL-53(Fe)/g-C_3_N_4_ photocatalysts was investigated by XRD and FT-IR analysis, and the results are presented in Figure 1. As shown in Figure 1a, the diffraction pattern of pure g-C_3_N_4_ exhibits two major peaks at 13.02° and 27.2°, corresponding to the (100), and (002) planes, respectively. When a small amount of MIL-53(Fe) was compounded with g-C_3_N_4_ (CM-5), the diffraction curve showed no evident change. With the further increase of MIL-53(Fe) content, the peaks related to g-C_3_N_4_ weakened gradually. At the same time, the characteristic peaks at 2θ of 9.1°, 12.6° attributed to (200), and (110) plane of MIL-53(Fe) (CCDC-690314) [37] appeared and shifted slightly to a low angle, which revealed an interaction between MIL-53(Fe) and g-C_3_N_4_. Moreover, the (11-1) crystalline plane at 17.6° [38] is almost invisible, indicating that the growth orientation of the crystalline planes is changed due to the compounding of g-C_3_N_4_, further indicating the successful synthesis of the MIL-53(Fe)/g-C_3_N_4_ composite. In addition, no diffraction peaks from impurities were identified.

In order to further identify the functional groups and analyze the molecular structure of MIL-53(Fe)/g-C_3_N_4_ samples, FT-IR spectroscopy was performed, and the results are shown in Figure 1b. As for g-C_3_N_4_, the multiple strong peaks in the range of 1200–1650 cm^−1^ can be assigned to the typical stretching modes of CN (C-N and C = N) heterocycles. The peaks at 1240, 1319 and 1418 cm^−1^ are ascribed to the aromatic C-N stretching vibration [39]. The peak at 806 cm^−1^ is assigned to the characteristic of triazine rings. The broad band at 2950–3600 cm^−1^ is attributed to the stretching vibration modes for the –NH and hydroxyl of the adsorbed water. After the addition of MIL-53(Fe), two extra adsorption peaks were observed. The peak at 553 cm^−1^ is attributed to the Fe-O bond between terephthalic acid and Fe^3+^ ions, and the C-H bonding vibration of benzene ring at 745 cm^−1^ is related to the organic linkers [40]. The appearance of peaks ascribed to MIL-53(Fe) also suggests the successful synthesis of the composite catalyst

#### 2.1.2. Morphology Characterization

**Figure 2:**
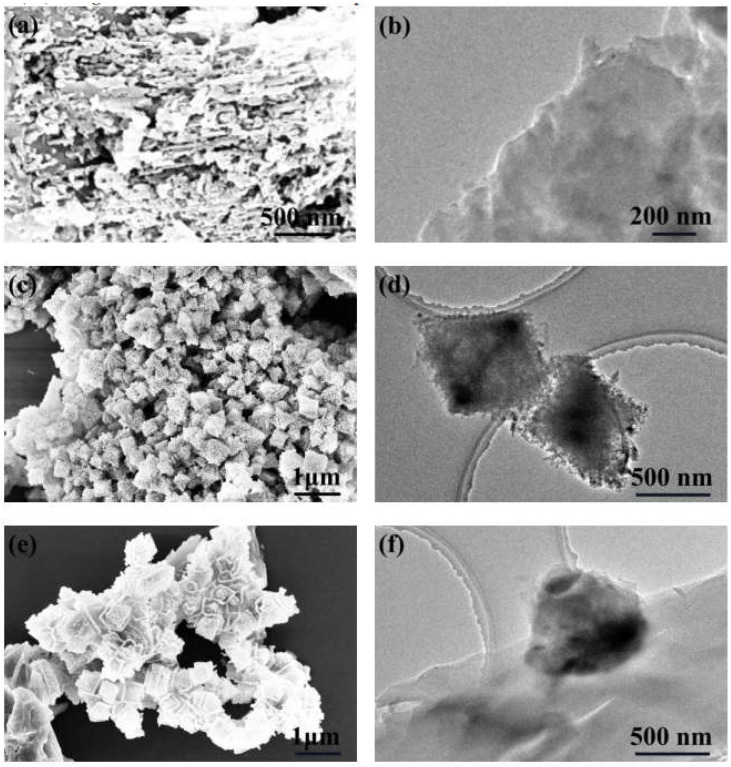
SEM and TEM images of (**a**,**b**) g-C_3_N_4_, (**c**,**d**) MIL-53(Fe) and (**e**,**f**) CM-10.

The morphologies of the MIL-53(Fe), g-C_3_N_4_, and CM-10 were characterized by SEM and TEM. Figure 2a,b show the as-prepared g-C_3_N_4_ sample has a multilayer lamellar structure. Pristine MIL-53(Fe) has an octahedral topology composed of nanoparticles with the diameter of 10–20 nm (Figure 2c,d). When MIL-53(Fe) was compounded with g-C_3_N_4_, the octahedron was dispersed densely and tightly between the g-C_3_N_4_ sheets, and the g-C_3_N_4_ nanolayers were wrapped closely on the surface of MIL-53(Fe), as shown in Figure 2e. The TEM image of CM-10 in Figure 2f further indicates the close contact between MIL-53(Fe) and g-C_3_N_4_. It is clear that the compounding can promote the separation of the layers and obtain a higher specific surface area (Figure S1). Additionally, the EDX element mapping images (Figure S2) clearly show that the CM-10 sample is composed of C, N, O, and Fe as expected. The uniform distribution of various elements further confirms that MIL-53(Fe) and g-C_3_N_4_ are fabricated into a composite.

#### 2.1.3. Optical Property Characterization

**Figure 3:**
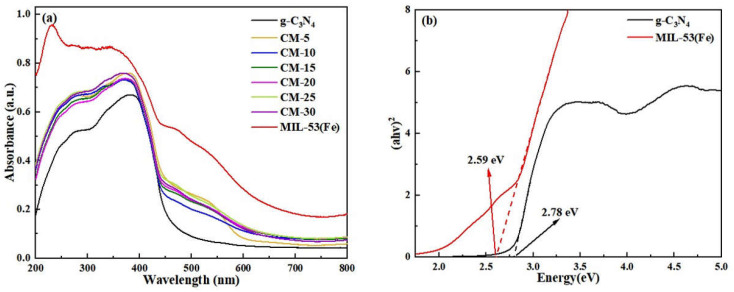
UV-visible diffuse reflectance spectra (**a**) and Tauc plots (**b**) of MIL-53(Fe)/g-C_3_N_4_ samples.

The UV-visible diffuse reflectance spectra (DRS) of g-C_3_N_4_, MIL-53(Fe), and a series of MIL-53(Fe)/g-C_3_N_4_ samples are depicted in Figure 3a. Absorption band edges at about 463 and 510 nm are displayed for g-C_3_N_4_ and MIL-53(Fe), respectively. Furthermore, the band gap energy (E_g_) of as-obtained samples was determined by drawing (αhν)^2^ against hν, where α, h, and ν, represent absorption coefficient, Planck constant, and light frequency, respectively. As illustrated in the inset of Figure 3b, the band gaps of pure g-C_3_N_4_ and MIL-53(Fe) are 2.78 eV and 2.59 eV, respectively. Compared with g-C_3_N_4_, MIL-53(Fe)/g-C_3_N_4_ composites exhibit an enhanced absorption in the range of visible light due to the wider absorption edge and higher absorption intensity of MIL-53(Fe), which is conducive to extending the light response range of the photocatalyst.

#### 2.1.4. XPS Characterization

**Figure 4:**
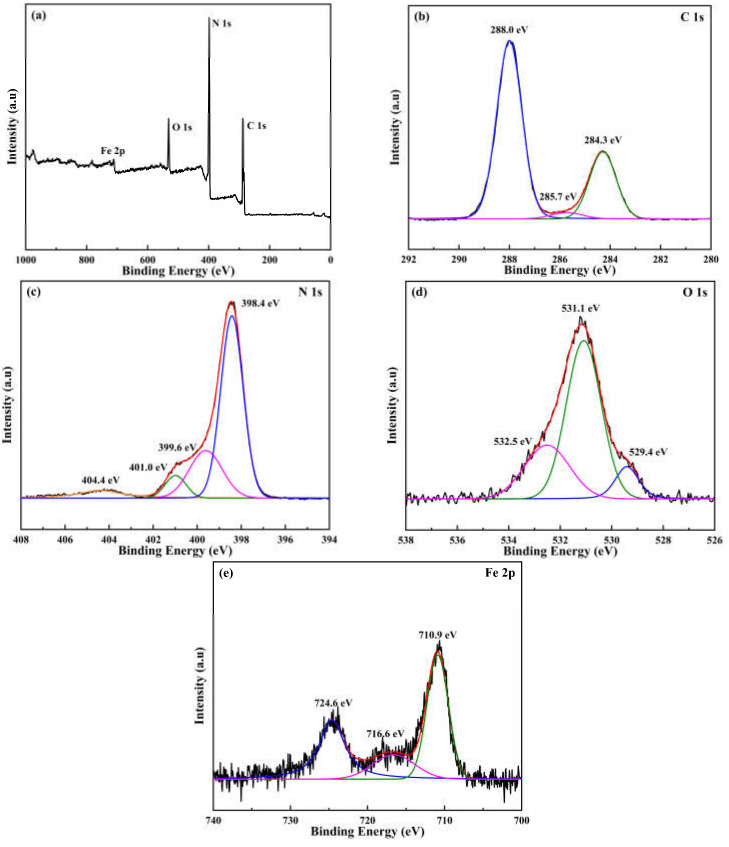
XPS spectra of CM-10: (**a**) full spectrum, (**b**) C 1s, (**c**) N 1s, (**d**) O 1s, and (**e**) Fe 2p.

XPS measurement was carried out to illustrate the chemical states and the surface chemical composition of the CM-10 photocatalyst. The XPS survey spectrum (Figure 4a) displays that the sample is composed of C, O, N and Fe elements. The C 1s spectrum in Figure 4b shows three binding energy peaks at 284.3, 285.7, and 288.0 eV. The peaks at 284.3 and 285.7 eV are attributed to sp^2^-hybridized C–C bond and sp^3^-bonded carbon (N-C) in the g-C_3_N_4_, respectively. The strong peak at 288.0 eV is ascribed to benzoic rings and C-O in the terephthalic linkers of MIL-53(Fe) and sp^2^-bonded carbon (N-C = N) in the triazine ring of g-C_3_N_4_ [41]. As for N 1s in Figure 4c, the peak at 398.4 eV is assigned to sp^2^-hybridized aromatic N bonded to carbon atoms (C = N-C). The weaker peaks at 399.6 and 401.0 eV correspond to the ternary nitrogen group N-(C)_3_ and amino groups C-NH_x_, respectively [42]. The peak at 404.4 eV can be attributed to Fe-N in the heterocycles. The high resolution XPS spectrum of O 1s in Figure 4d can be deconvoluted into three peaks. The weak peak at 529.4 eV can be attributed to the Fe-O bonds of MIL-53(Fe). The second peak at 531.1eV is related to the oxygen component of the H_2_BDC linkers. The peak at 532.5 eV corresponds to the adsorption oxygen species on the surface of the composite catalyst. Figure S3 shows the binding energies of Fe 2p_3/2_ and Fe 2p_1/2_ for pristine MIL-53(Fe) are 711.1 and 724.8 eV, respectively, and attributed to Fe^3+^ [43]. In addition, the peak at 717.0 eV is ascribed to the satellite peak of Fe 2p. For the CM-10 composite (Figure 4e), the corresponding binding energies of Fe 2p slightly shift to 724.6, 716.6, and 710.9 eV, indicating the lower electron cloud density and the chemical valence, which might be caused by an intimate interfacial contact and the formed Fe-N due to the interaction between MIL-53(Fe) and g-C_3_N_4_. The results further depict the formation of the CM-10 heterojunction photocatalyst.

#### 2.1.5. PL, EIS and PC Characterization

**Figure 5:**
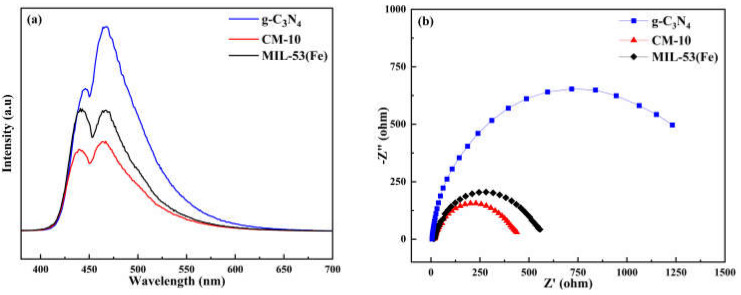
(**a**) Photoluminescence spectra and (**b**) electrochemical impedance spectra of g-C_3_N_4_, MIL-53(Fe) and CM-10 composite.

The separation efficiency of the photo-generated carriers of the as-synthesized g-C_3_N_4_, MIL-53(Fe), and the MIL-53(Fe)/g-C_3_N_4_ composite photocatalysts was studied by photoluminescence (PL) spectra (Figure S4 and Figure 5a). Generally, the PL peak with lower intensity represents the better separation efficiency and less recombination rate of photo-generated carriers. CM-10 composite displays a lowest PL intensity, revealing that the introduction of MIL-53(Fe) into g-C_3_N_4_ can inhibit the recombination of photo-generated electron-hole pairs, therefore enhancing the separation efficiency charge carriers. Furthermore, the electrochemical impedance spectroscopy (EIS)-Nyquist plots shown in Figure 5b further reflect a more efficient charge separation and transfer for the CM-10 sample with smaller semicircle diameter of Nyquist impedance plots. It implies CM-10 has smaller resistance during the photocatalytic process, which facilitates improving its photocatalytic activity.

**Figure 6:**
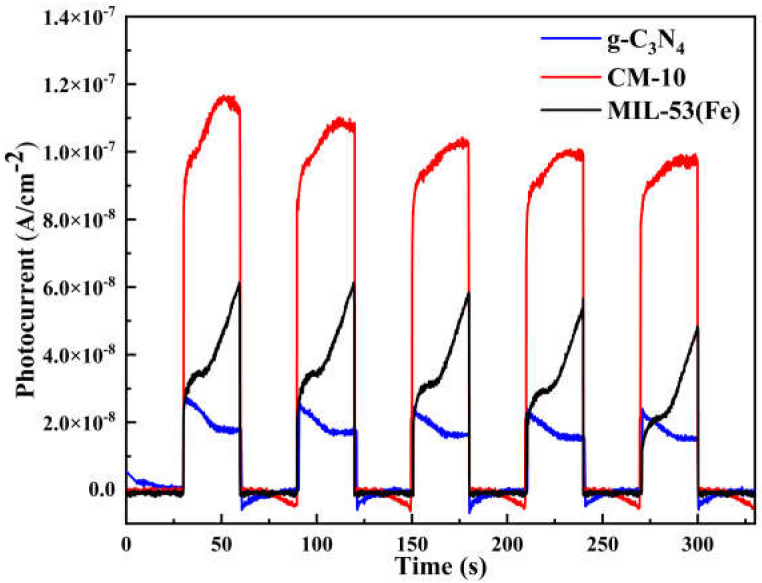
Transient photocurrent response of g-C_3_N_4_, MIL-53(Fe) and CM-10 composite.

Additionally, Figure 6 shows that the CM-10 exhibits the highest transient photocurrent response, which is about 1.9 and 4.2 times higher than that of pristine MIL-53(Fe) and g-C_3_N_4_, respectively. It indicates the outstanding capability of rapid separation and interfacial transfer of the charge carriers of the MIL-53(Fe)/g-C_3_N_4_ system.

### 2.2. Photocatalytic Performance

#### 2.2.1. The Effect of Incident Light

Table 1 lists the photocatalytic behavior of the CM-10 composite under different reaction conditions. In the blank test, no product was observed in the absence of photocatalyst or illumination, implying that a catalyst and light are indispensable for the photocatalytic oxidation of 5-HMF, and the effect of photolysis can be negligible. Under the illumination of a xenon lamp, the CM-10 composite photocatalyst can effectively converse 5-HMF to the target product DFF. As the incident light wavelength increased, the 5-HMF conversion declined gradually because of the reduced energy, whereas the trend of DFF selectivity was the reverse. The selectivity of DFF under UV light (>360 nm) is much lower than visible light (>420 nm), indicating that the high energy of UV light could result in the overoxidation of 5-HMF to FDCA.

#### 2.2.2. The Effect of MIL-53(Fe) Content

**Figure 7:**
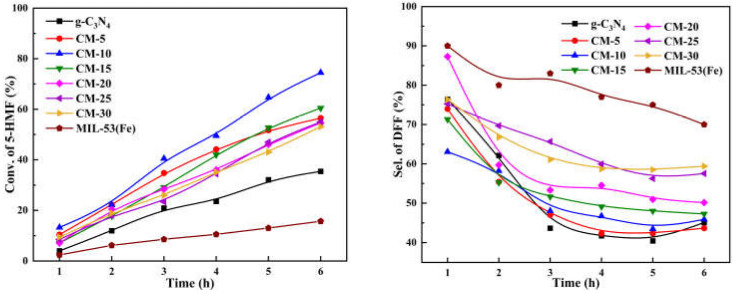
Photocatalytic behavior of different MIL-53(Fe)/g-C_3_N_4_ catalysts.

The photocatalytic behavior of the MIL-53(Fe)/g-C_3_N_4_ composites with different MIL-53(Fe) contents were evaluated for the aerobic selective oxidation of 5-HMF to DFF under the irradiation with O_2_ as an oxidant. Figure 7 shows the conversion of 5-HMF gradually increased with the reaction time, whereas the DFF yield increased first and then decreased. It can be ascribed that the excessive reaction time resulted in the deep oxidation of the target product DFF and a reduction of DFF selectivity. Pristine MIL-53(Fe) exhibits a low 5-HMF conversion of 15% with a high DFF selectivity of 71%. Compared with the reported compounds [25,26] used for the combining with g-C_3_N_4_, it exhibits a higher DFF selectivity due to following a different reaction mechanism, which is conducive to the increase of reaction performance of the as-synthesized MIL-53(Fe)/g-C_3_N_4_ composites. By contrast, g-C_3_N_4_ shows a higher 5-HMF conversion and lower DFF selectivity. With the increase of MIL-53(Fe) content, the selectivity of DFF increased gradually, whereas the conversion had a maximum value. The CM-10 composite exhibits the best photocatalytic performance. The catalysts with a higher MIL-53(Fe) content (CM-15, CM-20, CM-30) provide a decreased 5-HMF conversion and DFF yield, which is probably ascribed that a suitable MIL-53(Fe) concentration forms more contact interfaces and promotes carrier separation.

#### 2.2.3. The Effect of O_2_ Flow Rate

The effect of gas inflow on the photocatalytic oxidation of 5-HMF over CM-10 composite was also investigated. Table 2 shows that the photocatalytic activity increases gradually with the increase of oxygen concentration, which is attributed that electron transfer can be promoted by the introduction of more electrophilic species O_2_. When air was purged, the photocatalyst had a similar reaction performance due to an approximate oxygen concentration. By contrast, only trace 5-HMF was converted in presence of N_2_, which is ascribed to the existence of the dissolved oxygen in the reaction system. These results manifest that oxygen plays a critical role as an oxidant in this photocatalytic oxidation reaction.

#### 2.2.4. The Effect of Solvent

**Figure 8:**
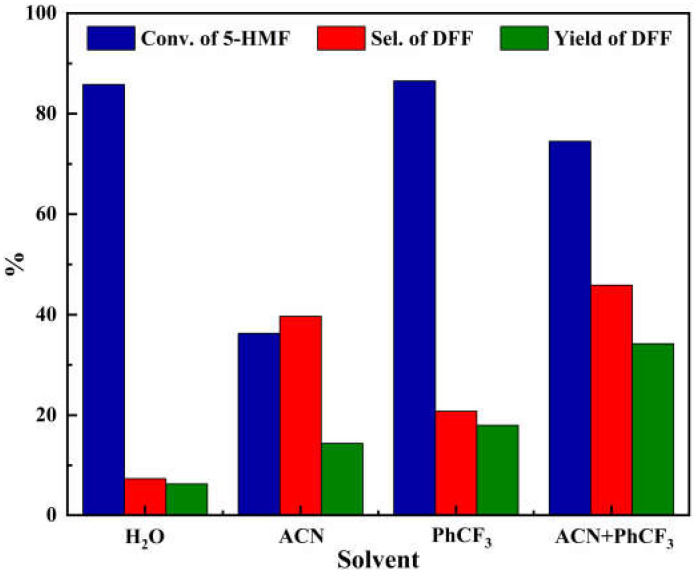
Photocatalytic performance of the CM-10 catalyst using different solvent.

To investigate the influence of the reaction solvent on the photocatalytic oxidation performance of 5-HMF to DFF, various solvents were used with the CM-10 catalyst and the results are shown in Figure 8. The obtained 5-HMF conversion in benzotrifluoride (PhCF_3_) solvent was higher than in other pure solvents, which is attributed to its lower polarity and higher ability to dissolve oxygen. In comparison, the polar solvents acetonitrile (ACN) resulted in a lower 5-HMF conversion probably due to the competition with the reactant for the active catalytic site. The highest selectivity to DFF was achieved using ACN as solvent, which might be ascribed to the lower electron withdrawing ability than other solvent. By using a mixture of ACN and PhCF_3_, the best photocatalytic performance can be acquired. In addition, the high conversion and low selectivity when using water as a solvent could be ascribed to a different active species and reaction route.

#### 2.2.5. Recycle Test

**Figure 9:**
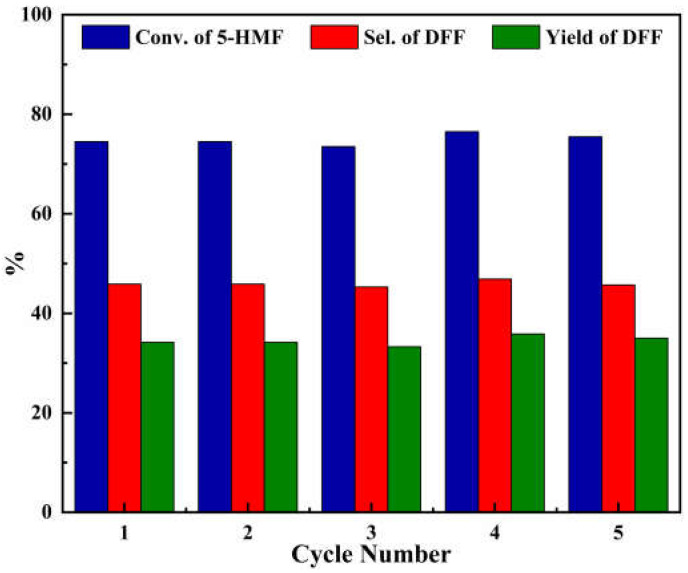
The recyclability of CM-10 for photocatalytic oxidation of 5-HMF.

The recyclability of CM-10 composite for the photocatalytic oxidation of 5-HMF to DFF was examined by recycling the catalyst under the same reaction conditions. After reaction completion, the used catalyst was recovered using centrifuge, washed with acetone, and dried at 60 °C. Figure 9 shows the CM-10 photocatalyst could be used up to five cycles without losing its activity, revealing its good stability and recyclability. The XPS spectra of the fresh and used catalysts (Figure S5) did not change remarkably, also indicating the stability of the catalyst.

### 2.3. Mechanism

#### 2.3.1. The Effect of Active Species

**Figure 10:**
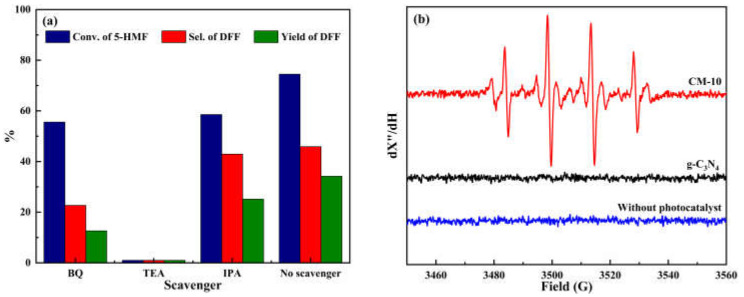
(**a**) Active species trapping experiment of CM-10 for photocatalytic oxidation of 5-HMF and (**b**) EPR spectra DMPO-·OH adduct in presence and absence of CM-10 and g-C_3_N_4_.

To investigate the active species involved in the photocatalytic selective oxidation of 5-HMF over the CM-10 catalyst, 1,4-benzoquinone (BQ), triethanolamine (TEA), and isopropyl alcohol (IPA) were added as the scavenger of ·O_2_^−^, h^+^, and ·OH, respectively. The results in Figure 10a reveal that the inhibitory effect follows the order TEA > BQ > IPA, which means that the importance of the reactive species in the photocatalytic oxidation of 5-HMF is h^+^ > ·O_2_^−^ > ·OH. In comparison, for g-C_3_N_4_ catalyst (Figure S6), only h^+^ and ·O_2_^−^ affected the reaction, whereas the addition of IPA did not change the reaction activity, suggesting that ·OH has no effect on the reaction.

To further compare the difference in the generation of hydroxyl radicals, EPR analysis was conducted in presence of 5,5-dimethyl-1-pyrroline N-oxide (DMPO) as a spin trap for hydroxyl radicals. As shown in Figure 10b, the EPR spectrum of CM-10 exhibits four lines with relative intensities of 1:2:2:1, again proving the existence of hydroxyl radicals.

#### 2.3.2. Possible Photocatalytic Mechanism

**Scheme 1:**
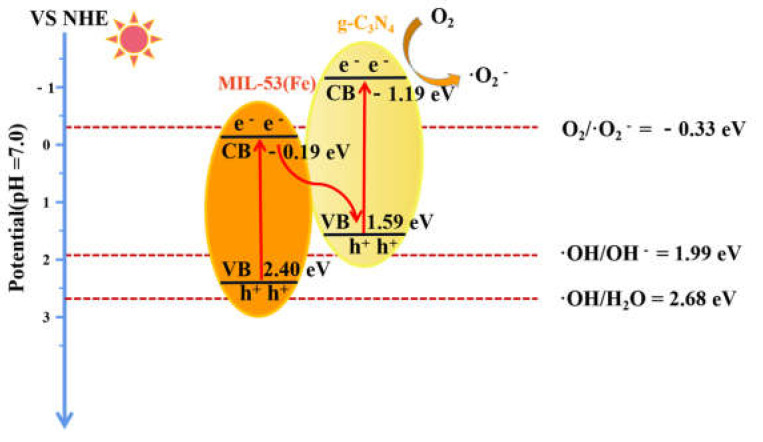
Photocatalytic mechanism diagram of CM-10 for the oxidation of 5-HMF.

In general, a semiconductor composites photocatalyst may follow either traditional type II or Z-scheme mechanism. In the case of MIL-53(Fe)/g-C_3_N_4_ composite, the CB and VB potentials of MIL-53(Fe) and g-C_3_N_4_ were determined by valence-band-X-ray photoelectron spectroscopy (VB-XPS) and DRS results. The results are described in Figure S7 and Scheme 1. The CB potential of MIL-53(Fe) (−0.19 eV) is positive compared with the O_2_/·O_2_^−^ potential (−0.33 eV) [44], and thus it is difficult to generate ·O_2_^−^ if following a traditional type-II mechanism. However, the trapping experiments revealed that ·O_2_^−^ is one of the main active species of the MIL-53(Fe)/g-C_3_N_4_ catalyst for the photocatalytic oxidation of 5-HMF. It can be speculated that the electron-hole separation of MIL-53(Fe)/g-C_3_N_4_ composite should follow a direct Z-scheme process. The photo-generated electrons (e^−^) on the CB of MIL-53(Fe) can combine with holes (h^+^) on VB of g-C_3_N_4_. Thereby, the electrons on the CB of g-C_3_N_4_ and the holes on the VB of MIL-53(Fe) can be effectively separated, and the recombination probability of photo-induced charge carriers is reduced sequentially. Moreover, the more positive VB edge (2.40 eV) of MIL-53(Fe) compared with g-C_3_N_4_ (1.59 eV) makes the MIL-53(Fe)/g-C_3_N_4_ composite possess better oxidation ability and exhibit higher performance for the photocatalytic oxidation of 5-HMF. In addition, the ESR results showed that ·OH can be generated on MIL-53(Fe)/g-C_3_N_4_ but not on g-C_3_N_4_ under light illumination. The quenching experimental results further manifest that the obtained ·OH on MIL-53(Fe)/g-C_3_N_4_ is one of the reactive species in the reaction, which facilitates the increase of photocatalytic oxidative performance.

Based on the above analysis, the reaction processes are speculated as follows:

MIL-53(Fe)/g-C_3_N_4_ + *hv* →MIL-53(Fe)(e^−^ + h^+^)/g-C_3_N_4_(e^−^ + h^+^) (1)

MIL-53(Fe)(e^−^ + h^+^)/g-C_3_N_4_(e^−^ + h^+^)→MIL-53(Fe)(h^+^)/g-C_3_N_4_(e^−^) (2)

e^−^ + O_2_→·O_2_^−^ (3)

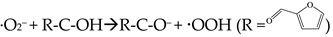
(4)

R-C-O^−^ + h^+^→R-C-O· (5)

R-C-O· + ·OOH→R-C=O + H_2_O_2_ (6)

R-C=O + H_2_O_2_→R-COOH (7)

Fe^3+^ + e^−^ →Fe^2+^ (8)

Fe^2+^ + H_2_O_2_→ Fe^3+^ + ·OH + OH^−^ (9)

·OH + R-C-OH→R-C-O·(10)

First, under irradiation, the photo-induced electron-hole pairs are generated (Equation (1)), transferred, and separated via a Z-scheme mechanism (Equation (2)). The electrons on the CB of g-C_3_N_4_ react with oxygen to produce ·O_2_^−^ (Equation (3)). The formed ·O_2_^−^ helps 5-HMF deprotonate alcohol hydroxyl into alkoxide anion (Equation (4)). It can react with the photo-generated hole by electron transfer to form the alkoxide radical (Equation (5)). The generated ·OOH from ·O_2_^−^ continues to react with the alkoxide radical, and finally, the corresponding aldehyde can be achieved (Equation (6)). The released H_2_O_2_ may further oxidize DFF to generate byproduct FDCA (Equation (7)). Simultaneously, part of the electrons on the CB of g-C_3_N_4_ can be transported to the Fe^3+^ clusters via interfacial charge transfer and reduce Fe^3+^ to Fe^2+^ due to the more negative E_CB_ of g-C_3_N_4_ compared with the redox potential of Fe^3+^/Fe^2+^ (0.77 eV) (Equation (8)). The obtained Fe^2+^ ions are unstable and react with hydrogen peroxide via a Fenton reaction to form Fe^3+^, accompanied by the generation of ·OH (Equation (9)) [45,46]. The formed ·OH with high reactivity can also oxidize alcohol hydroxyl (Equation (10)), and thus it enhances the photocatalytic activity and partially suppresses the overoxidation of DFF. The physical mixture of MIL-53(Fe) and g-C_3_N_4_ at a ratio of 10% exhibits low DFF selectivity (Figure S8), further revealing the importance of the generation of ·OH due to the heterojunction structure of MIL-53(Fe)/g-C_3_N_4_ composite.

## 3. Experimental

### 3.1. Chemicals

Melamine (C_3_H_6_N_6_), and Iron chloride hexahydrate (FeCl_3_.6H_2_O) were purchased from Macklin Biochemical Co., Ltd (Shanghai, China). N,N-Dimethylformamide (DMF), 2-Hydroxyterephthalic acid (H_2_BDC), and Ethanol absolute (C_2_H_6_O) were purchased from Sinopharm Chemical Reagent Co., Ltd (Shanghai, China) and used.

### 3.2. Catalyst Preparation

g-C_3_N_4_ was prepared via a polymerization method. In brief, 10 g of melamine was put into a crucible and calcinated in a muffle furnace at 550 °C for 4 h at a rate of 2.3 °C/min. After cooling down to room temperature, the obtained yellow solid was ground for use.

The typical preparation procedure of the MIL-53(Fe)/g-C_3_N_4_ photocatalysts was as follows: first, 2 mmol FeCl_3_.6H_2_O was dissolved in 56 mL DMF. Then, a certain amount g-C_3_N_4_ was added into the solution and suffered ultrasonic stirring for 30 min. After that, 2 mmol H_2_BDC was added and stirred for an additional 15 min. The resulting mixed solution was transferred to a stainless-steel autoclave and heated at 145 °C for 21 h. After cooling to room temperature, the obtained orange powder was washed with 20 mL of DMF and fresh ethanol several times, followed by stirring in 250 mL deionized water overnight. Finally, the resulting powder was dried at 120 °C under vacuum for 24 h. The obtained MIL-53(Fe)/g-C_3_N_4_ samples with different molar ratio of MIL-53(Fe) (5%, 10%, 15%, 20%, 25%, and 30%) were denoted as CM-5, CM-10, CM-15, CM-20, CM-25, and CM-30, respectively. For the sake of comparison, MIL-53(Fe) was also synthesized by the same procedure without g-C_3_N_4_.

### 3.3. Catalyst Characterization

The crystal phase and crystallinity of the catalysts were determined by using the powder X-ray diffraction (XRD. D8 Advance PW3040/6, Bruker, Germany) with Cu Kα radiation. The morphologies of samples were observed by a field emission scanning electron microscope (SEM. S-4800, Hitachi, Japan) at an accelerating voltage of 5 kV. Transmission electron microscopy (TEM. JEOL-2100F, Japan Inc., Tokyo, Japan) was conducted to further investigate the microstructure of catalyst at an accelerating voltage of 200 kV. The Brauner-Emmett-Teller (BET) specific surface areas of samples were determined by N_2_ absorption-desorption isotherms (JW-BK200, JWGB Sci & Tech Ltd., Beijing, China). The Fourier-transform infrared (FT-IR. NEXUS670, Nicolet, WI, USA) spectra of samples were collected with a resolution of 4 cm^−1^. The Ultraviolet-visible (UV-vis) diffuse reflectance spectra (DRS) were recorded using a UV−vis spectrometer (Cary 5000, Agilent, Malaysia) in the range of 200-800 nm, with BaSO_4_ for the corrected baseline. The X-ray photoelectron spectroscopy (XPS) was performed on an ESCLALAB 250Xi system (Thermo Fisher Scientific, Waltham, MA, USA) using Al Kα radiation. The photoluminescence (PL) spectra were measured on an FLA-980 spectrometer (Edinburgh Instrument, Livingston, UK) at room temperature. The photocurrent (PC) and electrochemical impedance spectroscopy (EIS) response measurement were performed using an electrochemical workstation (CHI 660E, CH Instruments Ins., Austin, TX, USA) with a standard three-electrode cell at room temperature. Electron paramagnetic resonance (EPR. Bruker, Billerica, UK) analysis was performed on a EMXplus-9.5/12 Spectrometer at X-band frequency of 9.85 GHz.

### 3.4. Photocatalytic Testing

The photocatalytic reactions were conducted in a two-necked flask (20 mL) under the irradiation of a 300 W xenon lamp at an O_2_ flow rate of 10 mL/min. In a typical run, 5-HMF (0.1 mmol) and 5 mL solvent were put into the flask. Then, 50 mg of the as-prepared MIL-53(Fe)/g-C_3_N_4_ catalyst was dispersed into the reactant solution under a magnetic stirring at the speed of 1000 rpm.

The reactant 5-HMF and products FFCA, DFF, and FDCA were analyzed by a high-performance liquid chromatography (HPLC. LC-20ADXR, Shimadzu, Japan) equipped with a diode array detector. These compounds were separated by a C18AQ column with a mobile phase consisting of 70% acetonitrile and 30% ultrapure water at a flow rate of 1 mL/min. The 5-HMF conversion, DFF yield, and DFF selectivity are evaluated according to Equations (11)–(13):

(11) $${\mathrm{Conversion}}_{HMF}=\frac{M^{0}{}_{HMF}-M_{HMF}}{M^{0}{}_{HMF}}\times 100\%$$

(12) $${\mathrm{Yield}}_{DFF}=\frac{M_{DFF}}{M^{0}{}_{HMF}}\times 100\%$$

(13) $${\mathrm{Selectivity}}_{DFF}=\frac{Yield_{DFF}}{{\mathrm{Conversion}}_{HMF}}\times 100\%$$

where $M^{0}{}_{HMF}$ is the molar amount of 5-HMF in the feedstock, and $M_{HMF}$ and $M_{DFF}$ are the molar amount of 5-HMF and DFF after the reaction, respectively.

## 4. Conclusions

In summary, the MIL-53(Fe)/g-C_3_N_4_ heterogeneous composite synthesized by a solvothermal method was employed for the photocatalytic aerobic oxidation of 5-HMF and exhibited good stability and recyclability. Compared with pure g-C_3_N_4_ and MIL-53(Fe), the MIL-53(Fe)/g-C_3_N_4_ composite displayed an improved photocatalytic performance, which can be ascribed to the formation of the heterogeneous structure and Z-scheme mechanism, facilitating the separation of photo-generated electron-hole pairs and the enhancement of oxidation ability. The interaction between the MIL-53(Fe) and g-C_3_N_4_ in the composite photocatalyst can promote the redox of Fe^3+^/Fe^2+^ and the generation of ·OH under light irradiation. ·O_2_^−^ and h^+^ species were also involved in the selective oxidation of 5-HMF. This paper has demonstrated the effectiveness of the MIL-53(Fe)/g-C_3_N_4_ composite photocatalyst and provided an inspiration on the catalyst design for the photocatalytic oxidation of biomass derivatives to value-added chemicals.

## Figures and Tables

**Table 1:** Photocatalytic behavior of CM-10 catalysts under incident light with different wavelength.

Entry	Conditions	Conv. of 5-HMF (%)	Sel. to DFF (%)	Yield to DFF (%)
1	No catalyst	0	0	0
2	Dark	0	0	0
3	λ > 360 nm	74.5	45.9	34.1
5	λ > 400 nm	29.5	59.6	17.6
6	λ > 420 nm	14.2	66.3	9.4

Reaction Conditions: Amount of 5-HMF: 0.0126 g (0.1 mmol); Reaction time: 6 h; Solvent: 3 mL ACN+2 mL PhCF_3_; O_2_ flow rate: 10 mL∙min^−1^.

**Table 2:** Photocatalytic behavior of CM-10 catalysts under different atmosphere.

Entry	Gas	Flow Rate (mL/min)	Conv. of 5-HMF (%)	Sel. to DFF (%)	Yield to DFF (%)
1	O_2_	2.5	45.9	19.8	9.1
2	O_2_	5	51.7	34.1	17.6
3	O_2_	10	74.5	45.9	34.1
4	O_2_	15	76.5	48.5	37.1
5	N_2_	10	4.3	31.5	1.4
6	Air	10	43.6	20.2	8.8

Reaction Conditions: Light wavelength: >360 nm; Amount of 5-HMF: 0.0126 g (0.1 mmol); Solvent: 3 mL ACN+2 mL PhCF_3_; Reaction time: 6 h.

## Data Availability

Not applicable.

## Supplementary Materials

The following are available online at https://www.mdpi.com/article/10.3390/molecules27238537/s1, Figure S1: Nitrogen adsorption–desorption isotherm and pore size distribution map of CM-10, MIL-53(Fe) and g-C_3_N_4_; Figure S2: EDS element mapping of CM-10; Figure S3: The XPS spectra of MIL-53(Fe); Figure S4: The PL spectra of a series of MIL-53(Fe)/g-C_3_N_4_ catalysts; Figure S5: The XPS spectra of the used CM-10 catalyst; Figure S6: Active species trapping experiment of g-C_3_N_4_ for photocatalytic oxidation of 5-HMF; Figure S7: VB XPS spectra of MIL-53(Fe) and g-C_3_N_4_; Figure S8: Photocatalytic performance of CM-10 and the physical mixture of 10% MIL-53(Fe) and g-C_3_N_4_.
