# Supplementary material for: Photocatalytic Aerobic Oxidation of Biomass-Derived 5-HMF to DFF over MIL-53(Fe)/g-C3N4 Composite
Source: Molecules. 2022 Dec 4;27(23):8537. doi: 10.3390/molecules27238537 (PMC9740462; doi:10.3390/molecules27238537)
Supplement: Supplementary file 1 [file molecules-27-08537-s001.zip › molecules-2008816-supplementary.pdf]

Photocatalytic aerobic oxidation of biomass-derived 5-HMF to DFF over MIL-53/g-C_3N_4 composite

Danyao Huang, Hao Wang, Ying Wu*

Key Laboratory of the Ministry of Education for Advanced Catalysis Materials, College of Chemistry and Life Science, Zhejiang Normal University, Jinhua 321004, China

*Corresponding author e-mail: yingwu@zjnu.cn

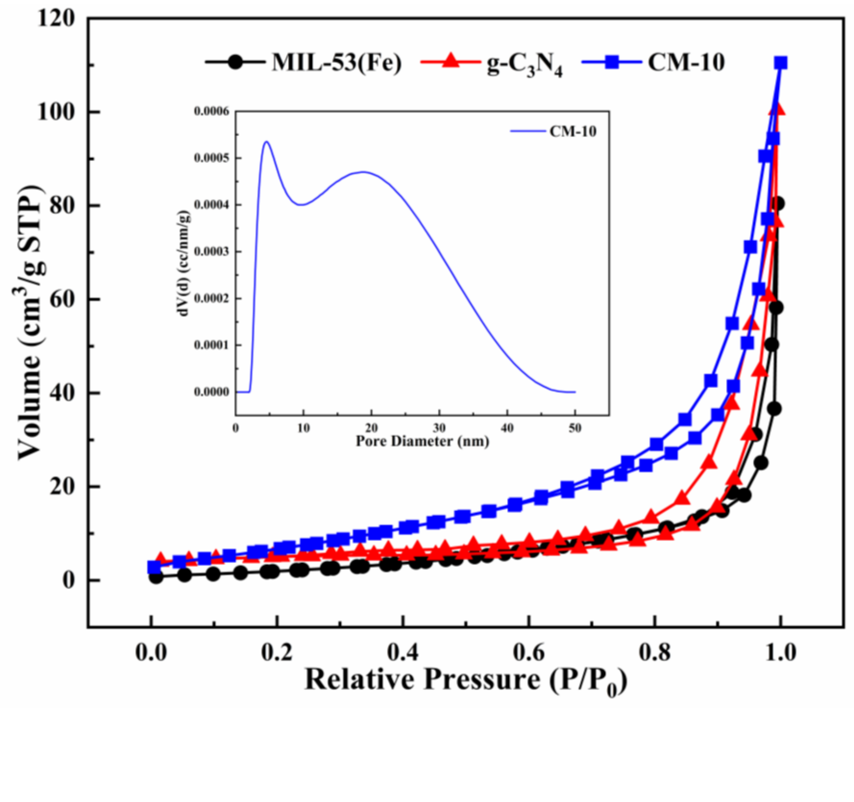

Figure S1. Nitrogen adsorption-desorption isotherm and pore size distribution map of CM-10, MIL-53(Fe) and g-C_3N_4.

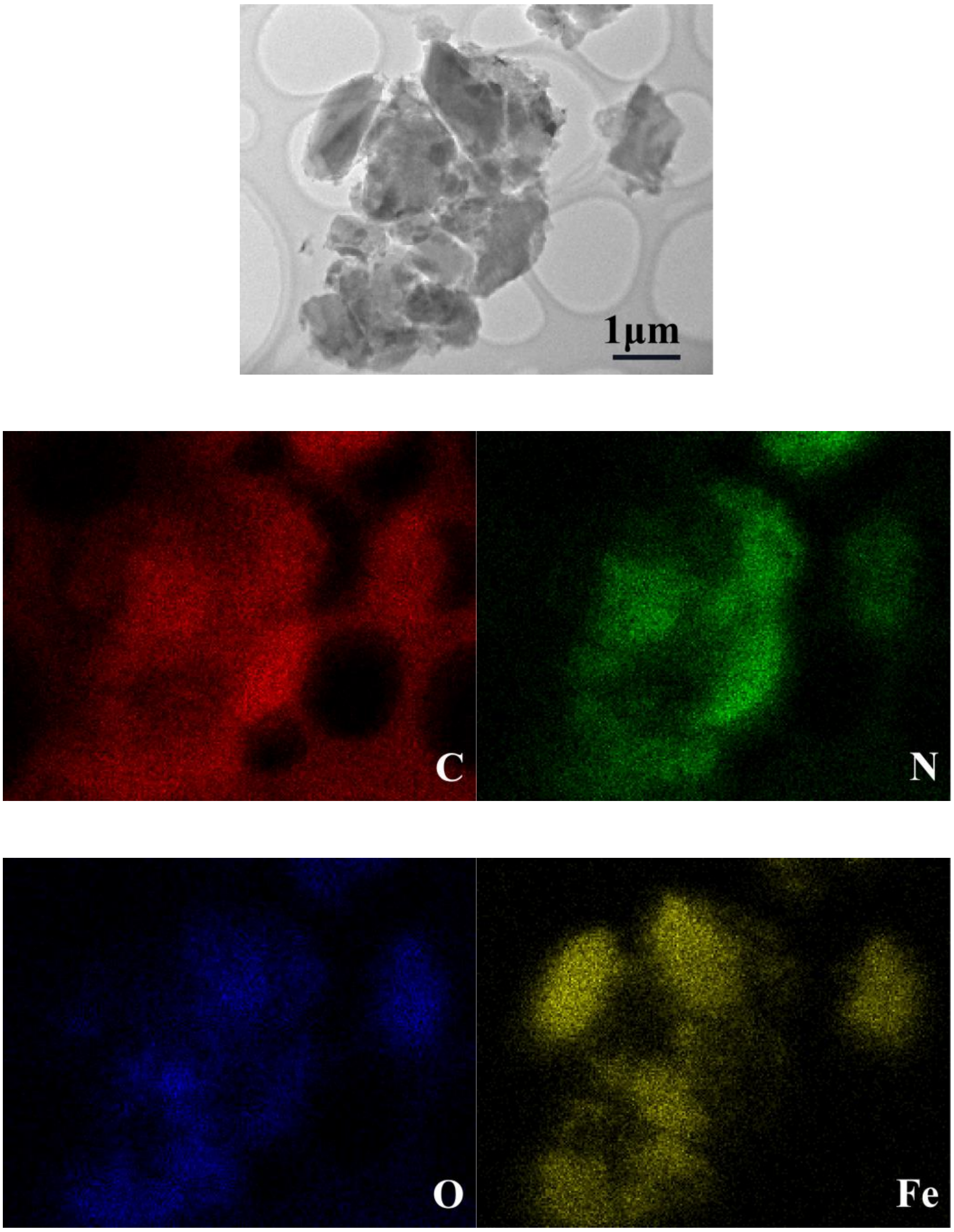

Figure S2. EDS element mapping of CM-10.

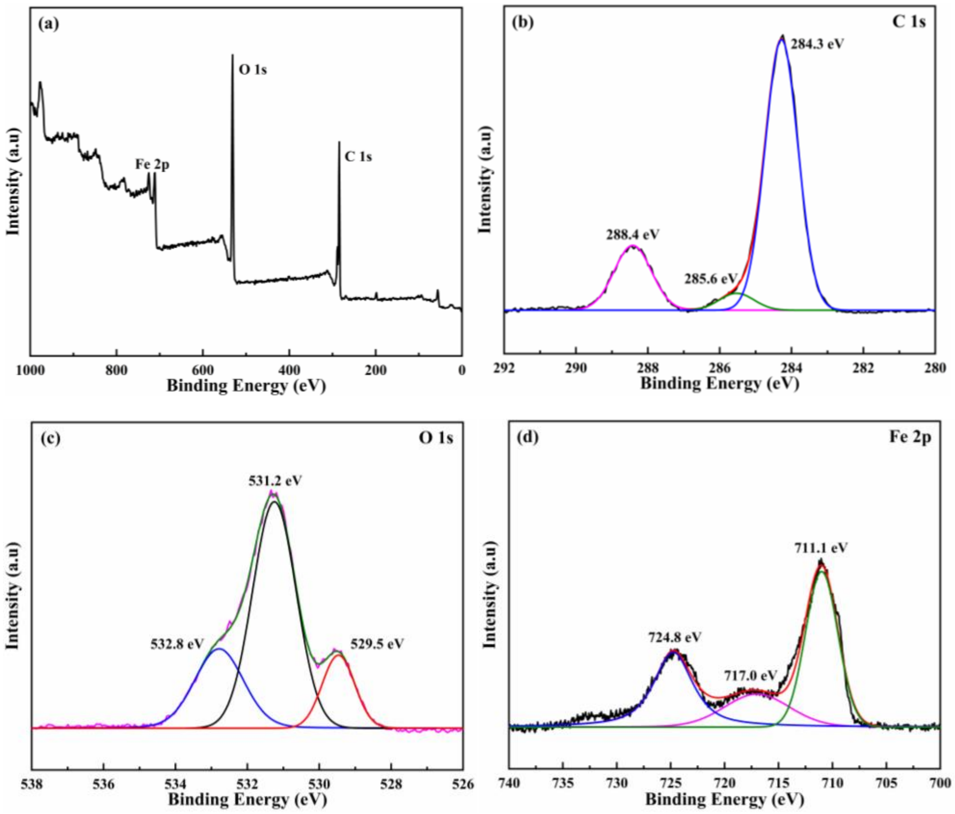

Figure S3. The XPS spectra of MIL-53(Fe).

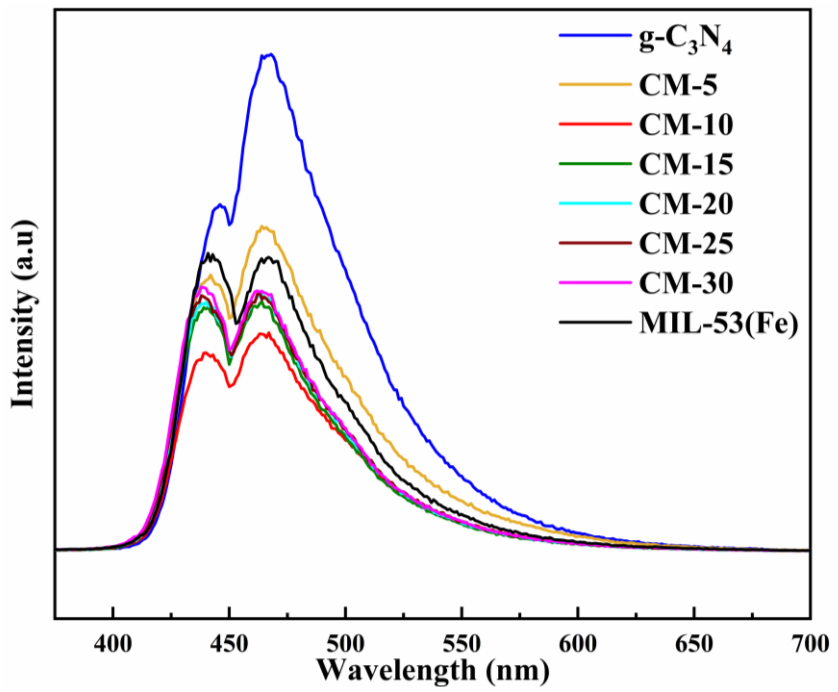

Figure S4. The PL spectra of a series of MIL-53(Fe)/g-C_3N_4 catalysts.

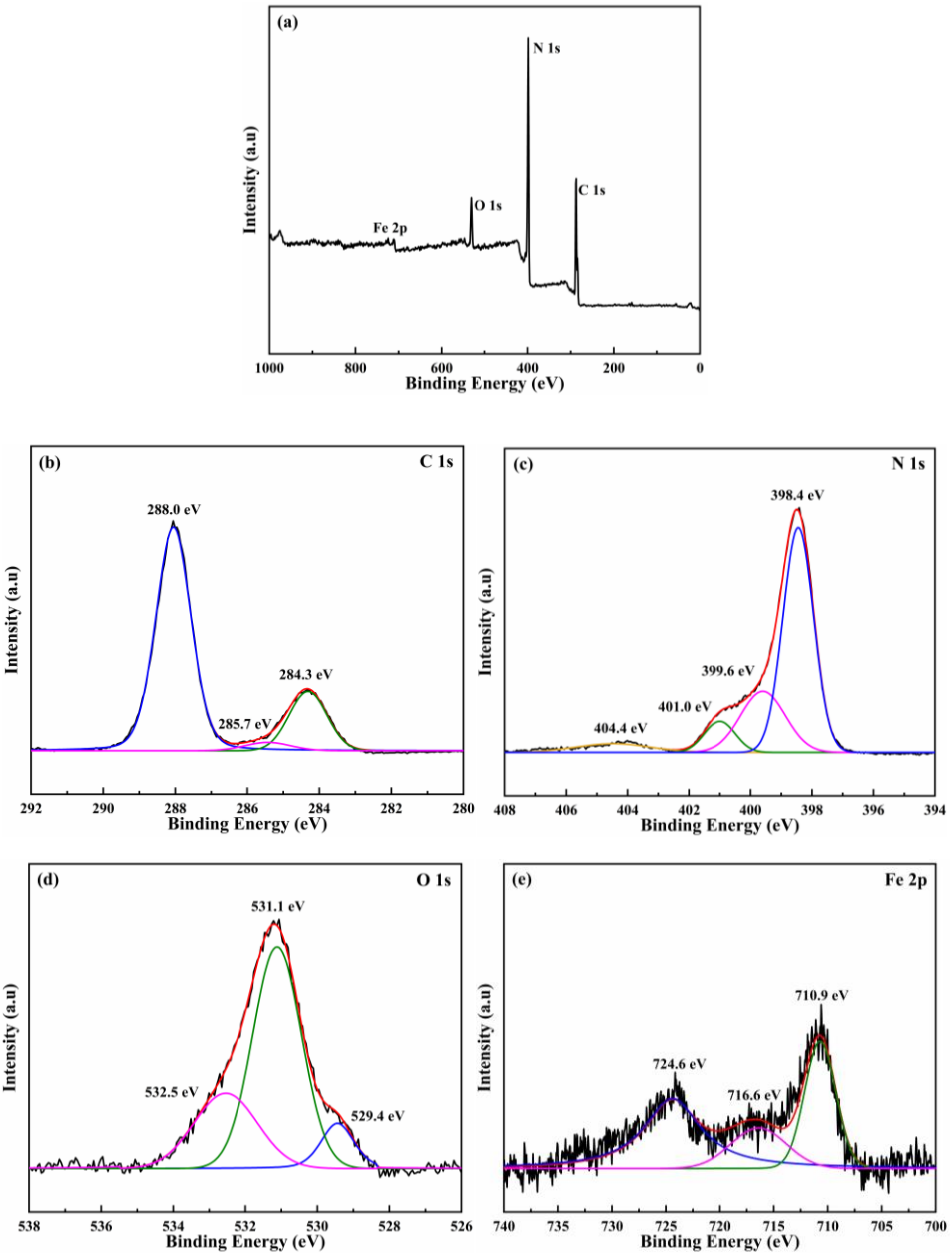

Figure S5. The XPS spectra of the used CM-10 catalysts.

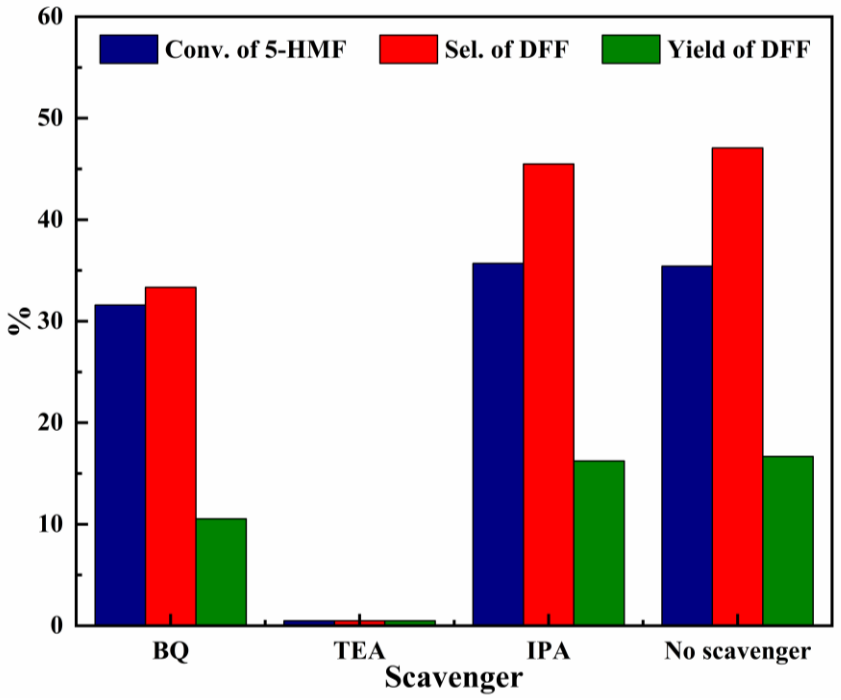

Figure S6. Active species trapping experiment of g-C_3N_4 for photocatalytic oxidation of 5-HMF.

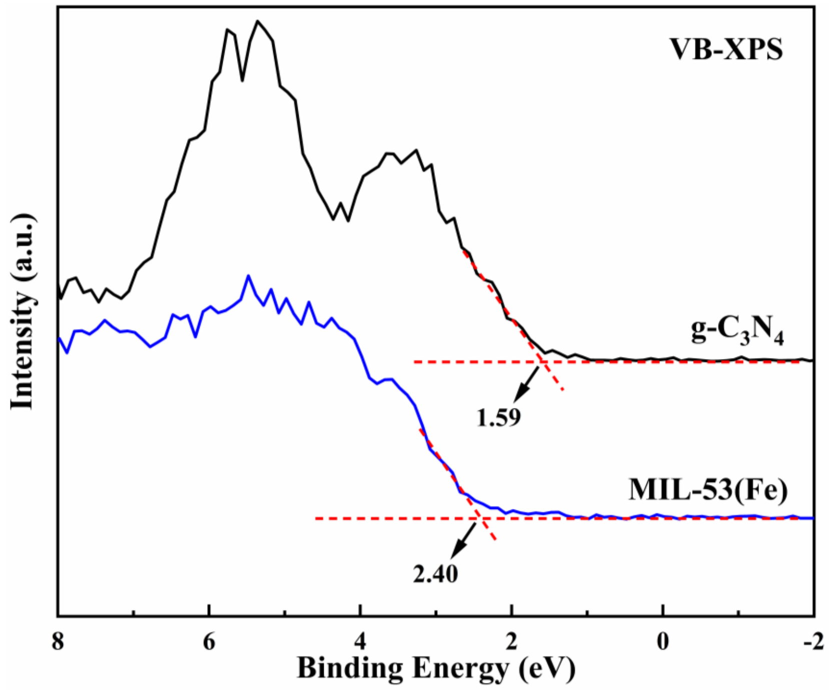

Figure S7. VB XPS spectra of MIL-53(Fe) and g-C_3N_4.

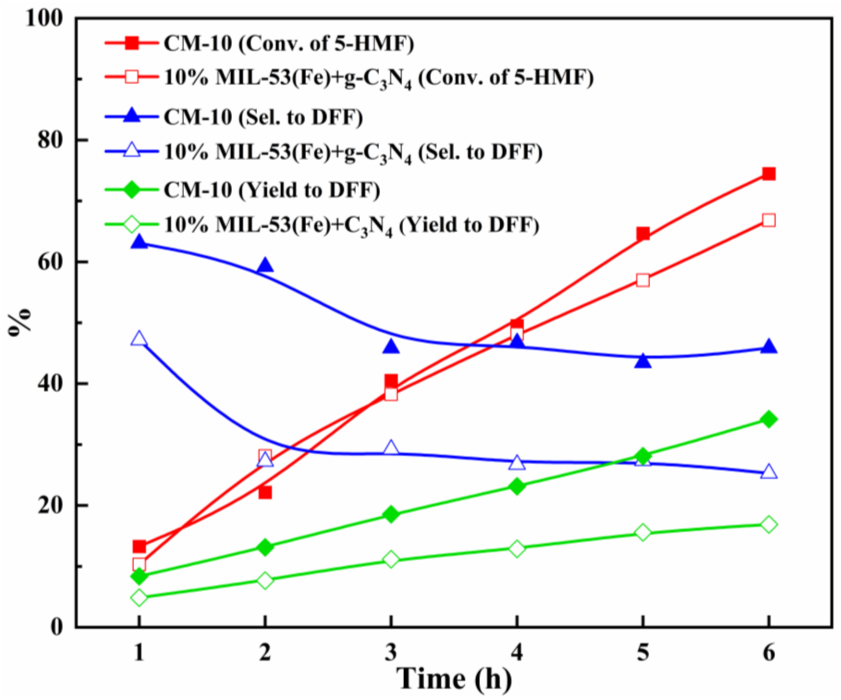

Figure S8. Photocatalytic performance of CM-10 and the physical mixture of 10% MIL-53(Fe) and g-C_3N_4.
